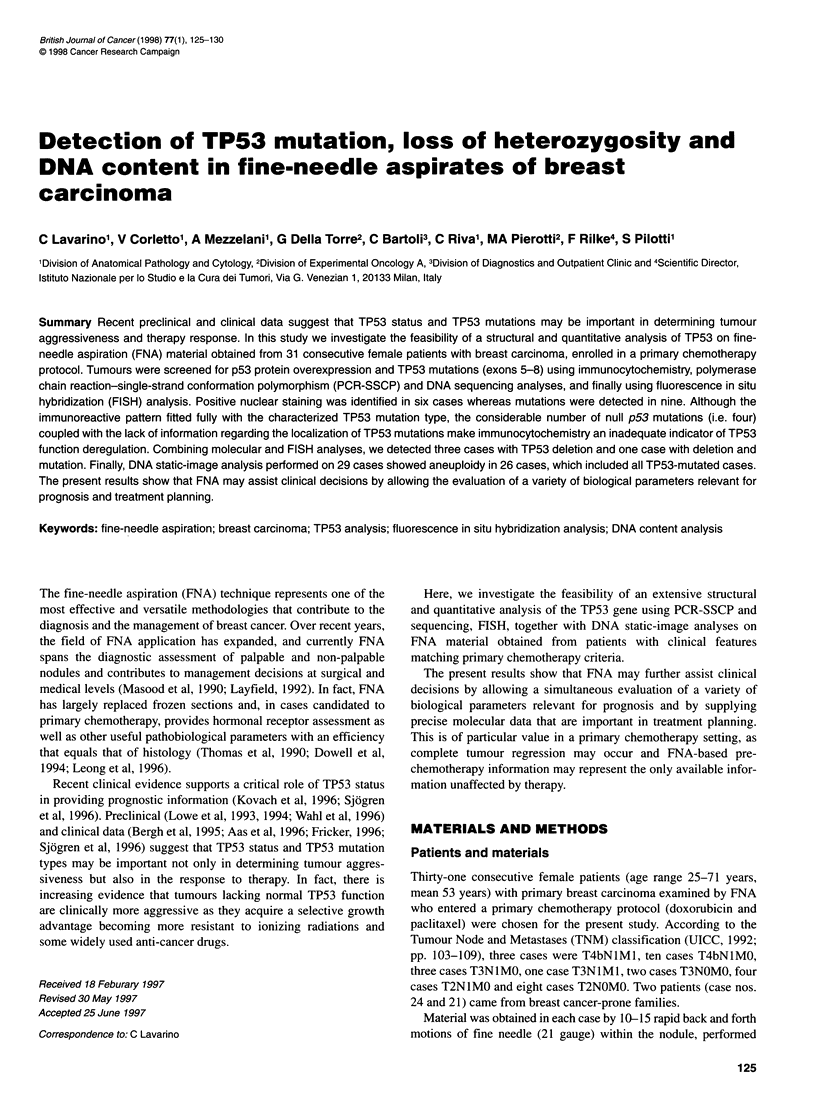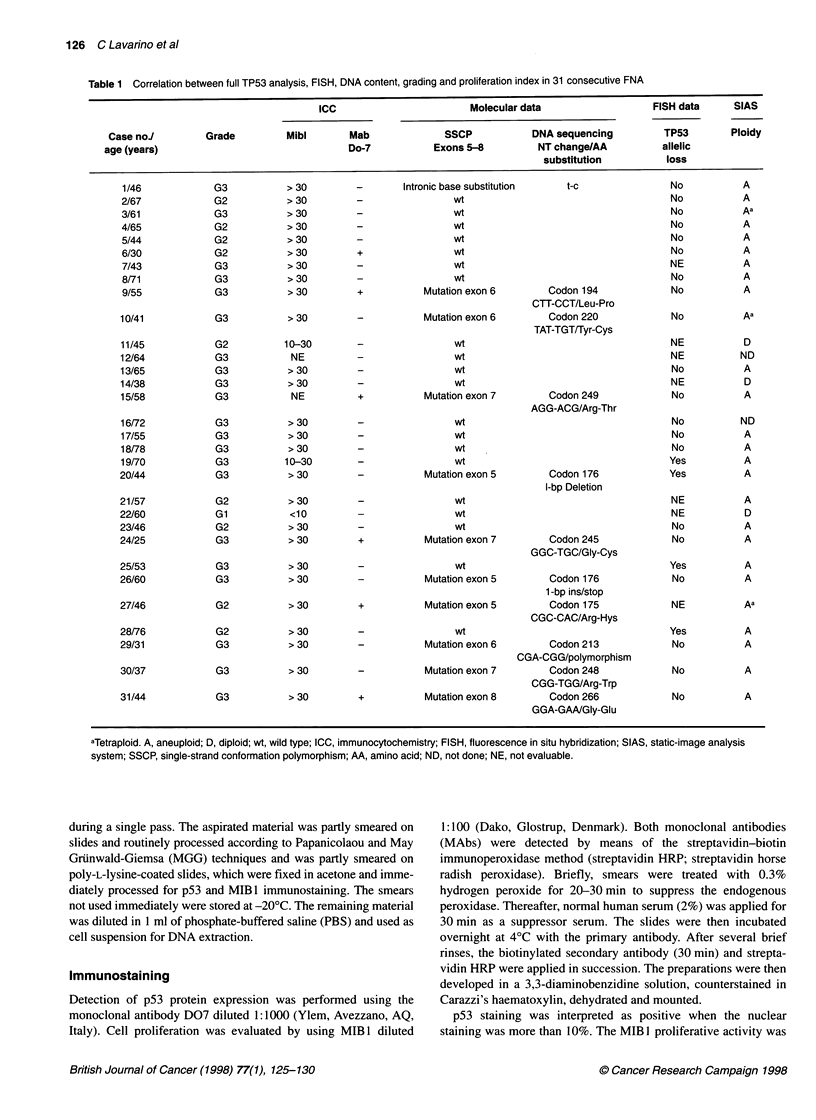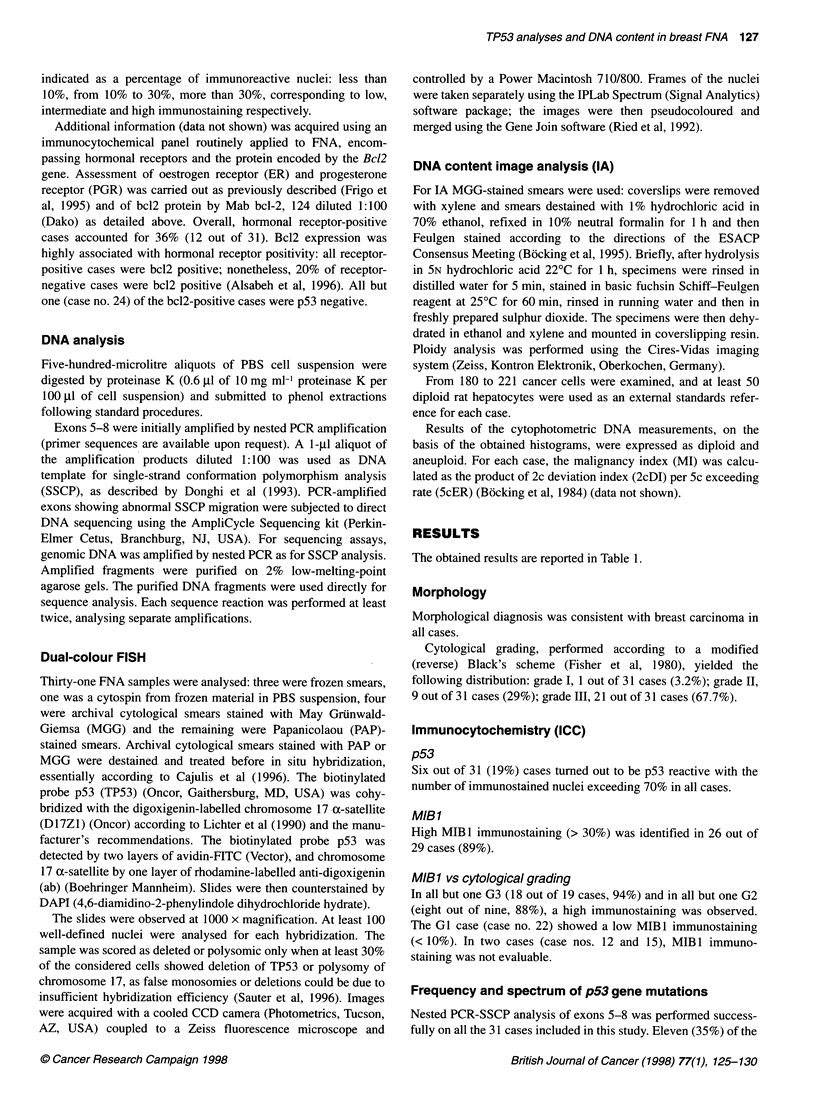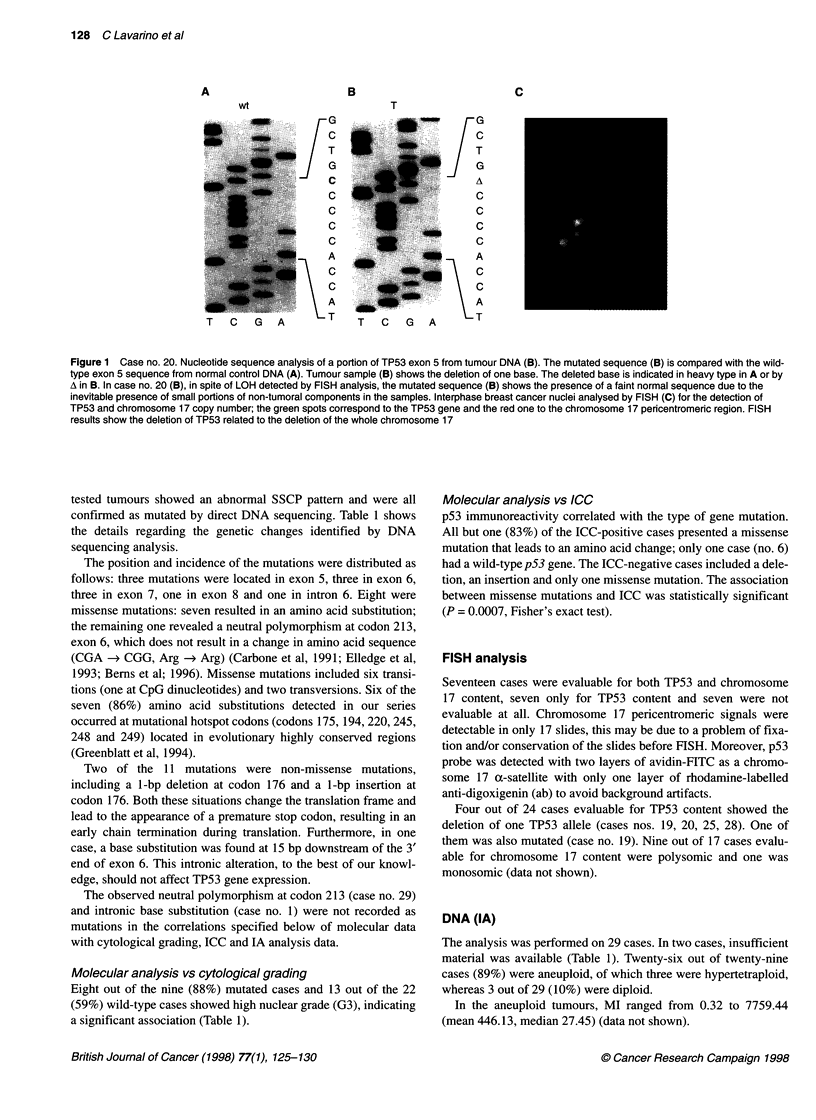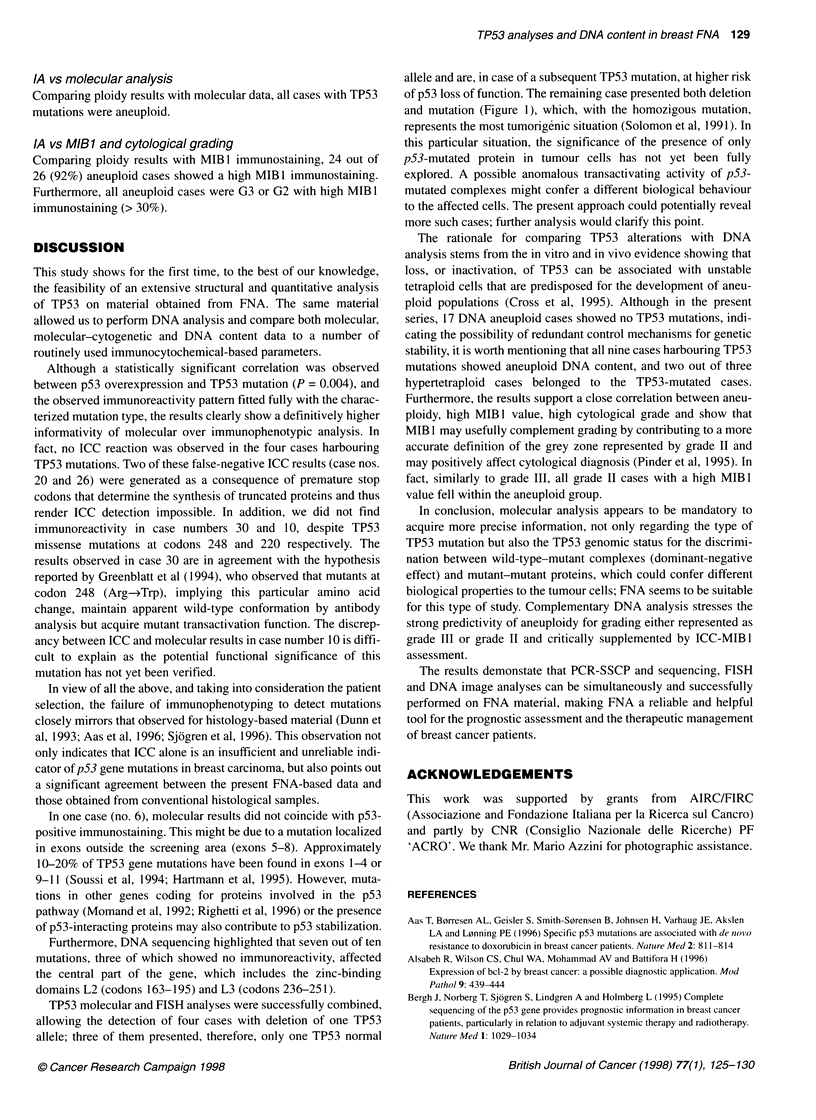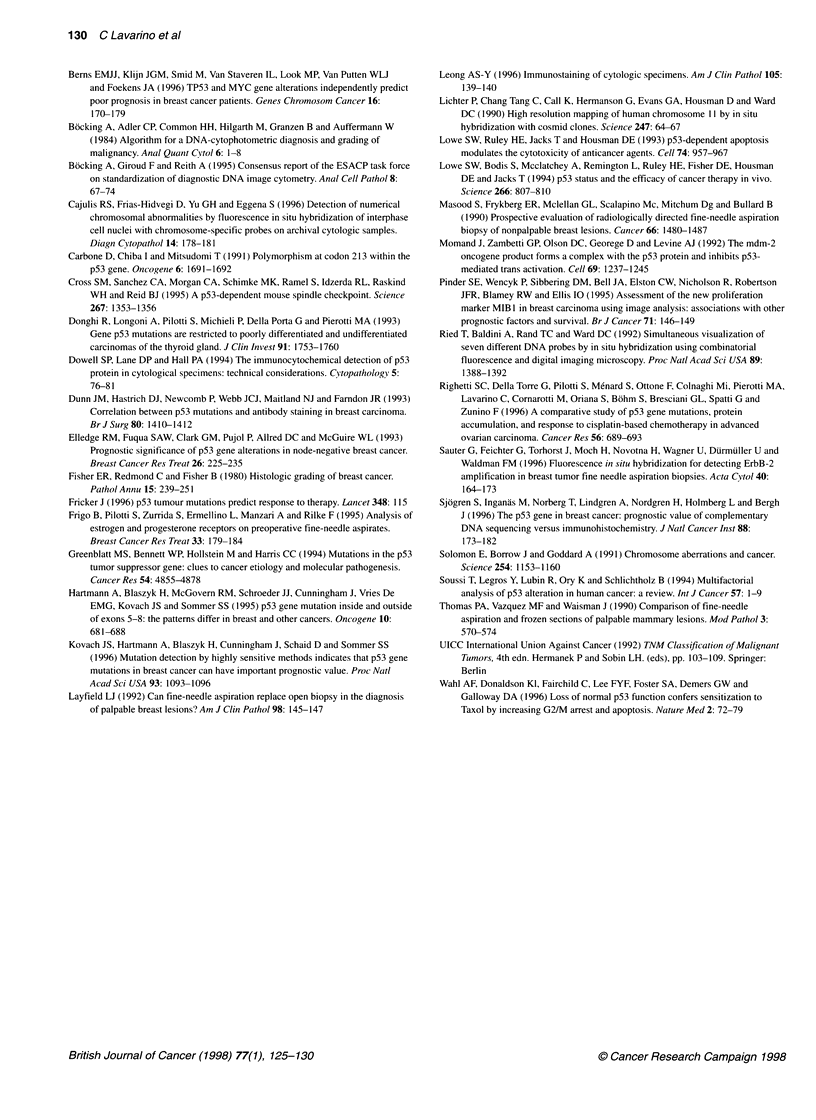# Detection of TP53 mutation, loss of heterozygosity and DNA content in fine-needle aspirates of breast carcinoma.

**DOI:** 10.1038/bjc.1998.20

**Published:** 1998

**Authors:** C. Lavarino, V. Corletto, A. Mezzelani, G. Della Torre, C. Bartoli, C. Riva, M. A. Pierotti, F. Rilke, S. Pilotti

**Affiliations:** Division of Anatomical Pathology and Cytology, Istituto Nazionale per lo Studio e la Cura dei Tumori, Milan, Italy.

## Abstract

**Images:**


British Journal of Cancer (1998) 77(1), 125–130
© 1998 Cancer Research Campaign

### Detection of TP53 mutation, loss of heterozygosity and DNA content in fine-needle aspirates of breast carcinoma

C Lavarino[1], V Corletto[1], A Mezzelani[1], G Della Torre[2], C Bartoli[3], C Riva[1], MA Pierotti[2], F Rilke[4], S Pilotti[1]

[1]Division of Anatomical Pathology and Cytology, [2]Division of Experimental Oncology A, [3]Division of Diagnostics and Outpatient Clinic and [4]Scientific Director, Istituto Nazionale per lo Studio e la Cura dei Tumori, Via G. Venezian 1, 20133 Milan, Italy

**Summary** Recent preclinical and clinical data suggest that TP53 status and TP53 mutations may be important in determining tumour aggressiveness and therapy response. In this study we investigate the feasibility of a structural and quantitative analysis of TP53 on fine-needle aspiration (FNA) material obtained from 31 consecutive female patients with breast carcinoma, enrolled in a primary chemotherapy protocol. Tumours were screened for p53 protein overexpression and TP53 mutations (exons 5–8) using immunocytochemistry, polymerase chain reaction–single-strand conformation polymorphism (PCR-SSCP) and DNA sequencing analyses, and finally using fluorescence in situ hybridization (FISH) analysis. Positive nuclear staining was identified in six cases whereas mutations were detected in nine. Although the immunoreactive pattern fitted fully with the characterized TP53 mutation type, the considerable number of null *p53* mutations (i.e. four) coupled with the lack of information regarding the localization of TP53 mutations make immunocytochemistry an inadequate indicator of TP53 function deregulation. Combining molecular and FISH analyses, we detected three cases with TP53 deletion and one case with deletion and mutation. Finally, DNA static-image analysis performed on 29 cases showed aneuploidy in 26 cases, which included all TP53-mutated cases. The present results show that FNA may assist clinical decisions by allowing the evaluation of a variety of biological parameters relevant for prognosis and treatment planning.

**Keywords:** fine-needle aspiration; breast carcinoma; TP53 analysis; fluorescence in situ hybridization analysis; DNA content analysis

The fine-needle aspiration (FNA) technique represents one of the most effective and versatile methodologies that contribute to the diagnosis and the management of breast cancer. Over recent years, the field of FNA application has expanded, and currently FNA spans the diagnostic assessment of palpable and non-palpable nodules and contributes to management decisions at surgical and medical levels (Masood et al, 1990; Layfield, 1992). In fact, FNA has largely replaced frozen sections and, in cases candidated to primary chemotherapy, provides hormonal receptor assessment as well as other useful pathobiological parameters with an efficiency that equals that of histology (Thomas et al, 1990; Dowell et al, 1994; Leong et al, 1996).

Recent clinical evidence supports a critical role of TP53 status in providing prognostic information (Kovach et al, 1996; Sjögren et al, 1996). Preclinical (Lowe et al, 1993, 1994; Wahl et al, 1996) and clinical data (Bergh et al, 1995; Aas et al, 1996; Fricker, 1996; Sjögren et al, 1996) suggest that TP53 status and TP53 mutation types may be important not only in determining tumour aggressiveness but also in the response to therapy. In fact, there is increasing evidence that tumours lacking normal TP53 function are clinically more aggressive as they acquire a selective growth advantage becoming more resistant to ionizing radiations and some widely used anti-cancer drugs.

*Received 18 Feburary 1997*
*Revised 30 May 1997*
*Accepted 25 June 1997*

*Correspondence to:* C Lavarino

Here, we investigate the feasibility of an extensive structural and quantitative analysis of the TP53 gene using PCR-SSCP and sequencing, FISH, together with DNA static-image analyses on FNA material obtained from patients with clinical features matching primary chemotherapy criteria.

The present results show that FNA may further assist clinical decisions by allowing a simultaneous evaluation of a variety of biological parameters relevant for prognosis and by supplying precise molecular data that are important in treatment planning. This is of particular value in a primary chemotherapy setting, as complete tumour regression may occur and FNA-based pre-chemotherapy information may represent the only available information unaffected by therapy.

### MATERIALS AND METHODS

### Patients and materials

Thirty-one consecutive female patients (age range 25–71 years, mean 53 years) with primary breast carcinoma examined by FNA who entered a primary chemotherapy protocol (doxorubicin and paclitaxel) were chosen for the present study. According to the Tumour Node and Metastases (TNM) classification (UICC, 1992; pp. 103–109), three cases were T4bN1M1, ten cases T4bN1M0, three cases T3N1M0, one case T3N1M1, two cases T3N0M0, four cases T2N1M0 and eight cases T2N0M0. Two patients (case nos. 24 and 21) came from breast cancer-prone families.

Material was obtained in each case by 10–15 rapid back and forth motions of fine needle (21 gauge) within the nodule, performed

**Table 1** Correlation between full TP53 analysis, FISH, DNA content, grading and proliferation index in 31 consecutive FNA

| Case no./ age (years) | Grade | ICC | | Molecular data | | FISH data | SIAS |
|---|---|---|---|---|---|---|---|
| | | Mibl | Mab Do-7 | SSCP Exons 5–8 | DNA sequencing NT change/AA substitution | TP53 allelic loss | Ploidy |
| 1/46 | G3 | > 30 | – | Intronic base substitution | t-c | No | A |
| 2/67 | G2 | > 30 | – | wt | | No | A |
| 3/61 | G3 | > 30 | – | wt | | No | A[a] |
| 4/65 | G2 | > 30 | – | wt | | No | A |
| 5/44 | G2 | > 30 | – | wt | | No | A |
| 6/30 | G2 | > 30 | + | wt | | No | A |
| 7/43 | G3 | > 30 | – | wt | | NE | A |
| 8/71 | G3 | > 30 | – | wt | | No | A |
| 9/55 | G3 | > 30 | + | Mutation exon 6 | Codon 194 CTT-CCT/Leu-Pro | No | A |
| 10/41 | G3 | > 30 | – | Mutation exon 6 | Codon 220 TAT-TGT/Tyr-Cys | No | A[a] |
| 11/45 | G2 | 10–30 | – | wt | | NE | D |
| 12/64 | G3 | NE | – | wt | | NE | ND |
| 13/65 | G3 | > 30 | – | wt | | No | A |
| 14/38 | G3 | > 30 | – | wt | | NE | D |
| 15/58 | G3 | NE | + | Mutation exon 7 | Codon 249 AGG-ACG/Arg-Thr | No | A |
| 16/72 | G3 | > 30 | – | wt | | No | ND |
| 17/55 | G3 | > 30 | – | wt | | No | A |
| 18/78 | G3 | > 30 | – | wt | | No | A |
| 19/70 | G3 | 10–30 | – | wt | | Yes | A |
| 20/44 | G3 | > 30 | – | Mutation exon 5 | Codon 176 l-bp Deletion | Yes | A |
| 21/57 | G2 | > 30 | – | wt | | NE | A |
| 22/60 | G1 | <10 | – | wt | | NE | D |
| 23/46 | G2 | > 30 | – | wt | | No | A |
| 24/25 | G3 | > 30 | + | Mutation exon 7 | Codon 245 GGC-TGC/Gly-Cys | No | A |
| 25/53 | G3 | > 30 | – | wt | | Yes | A |
| 26/60 | G3 | > 30 | – | Mutation exon 5 | Codon 176 1-bp ins/stop | No | A |
| 27/46 | G2 | > 30 | + | Mutation exon 5 | Codon 175 CGC-CAC/Arg-Hys | NE | A[a] |
| 28/76 | G2 | > 30 | – | wt | | Yes | A |
| 29/31 | G3 | > 30 | – | Mutation exon 6 | Codon 213 CGA-CGG/polymorphism | No | A |
| 30/37 | G3 | > 30 | – | Mutation exon 7 | Codon 248 CGG-TGG/Arg-Trp | No | A |
| 31/44 | G3 | > 30 | + | Mutation exon 8 | Codon 266 GGA-GAA/Gly-Glu | No | A |

[a]Tetraploid. A, aneuploid; D, diploid; wt, wild type; ICC, immunocytochemistry; FISH, fluorescence in situ hybridization; SIAS, static-image analysis system; SSCP, single-strand conformation polymorphism; AA, amino acid; ND, not done; NE, not evaluable.

during a single pass. The aspirated material was partly smeared on slides and routinely processed according to Papanicolaou and May Grünwald-Giemsa (MGG) techniques and was partly smeared on poly-L-lysine-coated slides, which were fixed in acetone and immediately processed for p53 and MIB1 immunostaining. The smears not used immediately were stored at –20°C. The remaining material was diluted in 1 ml of phosphate-buffered saline (PBS) and used as cell suspension for DNA extraction.

### Immunostaining

Detection of p53 protein expression was performed using the monoclonal antibody DO7 diluted 1:1000 (Ylem, Avezzano, AQ, Italy). Cell proliferation was evaluated by using MIB1 diluted 1:100 (Dako, Glostrup, Denmark). Both monoclonal antibodies (MAbs) were detected by means of the streptavidin–biotin immunoperoxidase method (streptavidin HRP; streptavidin horse radish peroxidase). Briefly, smears were treated with 0.3% hydrogen peroxide for 20–30 min to suppress the endogenous peroxidase. Thereafter, normal human serum (2%) was applied for 30 min as a suppressor serum. The slides were then incubated overnight at 4°C with the primary antibody. After several brief rinses, the biotinylated secondary antibody (30 min) and streptavidin HRP were applied in succession. The preparations were then developed in a 3,3-diaminobenzidine solution, counterstained in Carazzi's haematoxylin, dehydrated and mounted.

p53 staining was interpreted as positive when the nuclear staining was more than 10%. The MIB1 proliferative activity was

© Cancer Research Campaign 1998

indicated as a percentage of immunoreactive nuclei: less than 10%, from 10% to 30%, more than 30%, corresponding to low, intermediate and high immunostaining respectively.

Additional information (data not shown) was acquired using an immunocytochemical panel routinely applied to FNA, encompassing hormonal receptors and the protein encoded by the *Bcl2* gene. Assessment of oestrogen receptor (ER) and progesterone receptor (PGR) was carried out as previously described (Frigo et al, 1995) and of bcl2 protein by Mab bcl-2, 124 diluted 1:100 (Dako) as detailed above. Overall, hormonal receptor-positive cases accounted for 36% (12 out of 31). Bcl2 expression was highly associated with hormonal receptor positivity: all receptor-positive cases were bcl2 positive; nonetheless, 20% of receptor-negative cases were bcl2 positive (Alsabeh et al, 1996). All but one (case no. 24) of the bcl2-positive cases were p53 negative.

### DNA analysis

Five-hundred-microlitre aliquots of PBS cell suspension were digested by proteinase K (0.6 μl of 10 mg $ml^{-1}$ proteinase K per 100 μl of cell suspension) and submitted to phenol extractions following standard procedures.

Exons 5–8 were initially amplified by nested PCR amplification (primer sequences are available upon request). A 1-μl aliquot of the amplification products diluted 1:100 was used as DNA template for single-strand conformation polymorphism analysis (SSCP), as described by Donghi et al (1993). PCR-amplified exons showing abnormal SSCP migration were subjected to direct DNA sequencing using the AmpliCycle Sequencing kit (Perkin-Elmer Cetus, Branchburg, NJ, USA). For sequencing assays, genomic DNA was amplified by nested PCR as for SSCP analysis. Amplified fragments were purified on 2% low-melting-point agarose gels. The purified DNA fragments were used directly for sequence analysis. Each sequence reaction was performed at least twice, analysing separate amplifications.

### Dual-colour FISH

Thirty-one FNA samples were analysed: three were frozen smears, one was a cytospin from frozen material in PBS suspension, four were archival cytological smears stained with May Grünwald-Giemsa (MGG) and the remaining were Papanicolaou (PAP)-stained smears. Archival cytological smears stained with PAP or MGG were destained and treated before in situ hybridization, essentially according to Cajulis et al (1996). The biotinylated probe p53 (TP53) (Oncor, Gaithersburg, MD, USA) was cohybridized with the digoxigenin-labelled chromosome 17 α-satellite (D17Z1) (Oncor) according to Lichter et al (1990) and the manufacturer's recommendations. The biotinylated probe p53 was detected by two layers of avidin-FITC (Vector), and chromosome 17 α-satellite by one layer of rhodamine-labelled anti-digoxigenin (ab) (Boehringer Mannheim). Slides were then counterstained by DAPI (4,6-diamidino-2-phenylindole dihydrochloride hydrate).

The slides were observed at 1000 × magnification. At least 100 well-defined nuclei were analysed for each hybridization. The sample was scored as deleted or polysomic only when at least 30% of the considered cells showed deletion of TP53 or polysomy of chromosome 17, as false monosomies or deletions could be due to insufficient hybridization efficiency (Sauter et al, 1996). Images were acquired with a cooled CCD camera (Photometrics, Tucson, AZ, USA) coupled to a Zeiss fluorescence microscope and controlled by a Power Macintosh 710/800. Frames of the nuclei were taken separately using the IPLab Spectrum (Signal Analytics) software package; the images were then pseudocoloured and merged using the Gene Join software (Ried et al, 1992).

### DNA content image analysis (IA)

For IA MGG-stained smears were used: coverslips were removed with xylene and smears destained with 1% hydrochloric acid in 70% ethanol, refixed in 10% neutral formalin for 1 h and then Feulgen stained according to the directions of the ESACP Consensus Meeting (Böcking et al, 1995). Briefly, after hydrolysis in 5N hydrochloric acid 22°C for 1 h, specimens were rinsed in distilled water for 5 min, stained in basic fuchsin Schiff–Feulgen reagent at 25°C for 60 min, rinsed in running water and then in freshly prepared sulphur dioxide. The specimens were then dehydrated in ethanol and xylene and mounted in coverslipping resin. Ploidy analysis was performed using the Cires-Vidas imaging system (Zeiss, Kontron Elektronik, Oberkochen, Germany).

From 180 to 221 cancer cells were examined, and at least 50 diploid rat hepatocytes were used as an external standards reference for each case.

Results of the cytophotometric DNA measurements, on the basis of the obtained histograms, were expressed as diploid and aneuploid. For each case, the malignancy index (MI) was calculated as the product of 2c deviation index (2cDI) per 5c exceeding rate (5cER) (Böcking et al, 1984) (data not shown).

### RESULTS

The obtained results are reported in Table 1.

### Morphology

Morphological diagnosis was consistent with breast carcinoma in all cases.

Cytological grading, performed according to a modified (reverse) Black's scheme (Fisher et al, 1980), yielded the following distribution: grade I, 1 out of 31 cases (3.2%); grade II, 9 out of 31 cases (29%); grade III, 21 out of 31 cases (67.7%).

### Immunocytochemistry (ICC)

### *p53*

Six out of 31 (19%) cases turned out to be p53 reactive with the number of immunostained nuclei exceeding 70% in all cases.

### *MIB1*

High MIB1 immunostaining (> 30%) was identified in 26 out of 29 cases (89%).

### *MIB1 vs cytological grading*

In all but one G3 (18 out of 19 cases, 94%) and in all but one G2 (eight out of nine, 88%), a high immunostaining was observed. The G1 case (case no. 22) showed a low MIB1 immunostaining (< 10%). In two cases (case nos. 12 and 15), MIB1 immunostaining was not evaluable.

### Frequency and spectrum of *p53* gene mutations

Nested PCR-SSCP analysis of exons 5–8 was performed successfully on all the 31 cases included in this study. Eleven (35%) of the

© Cancer Research Campaign 1998

**Figure 1** Case no. 20. Nucleotide sequence analysis of a portion of TP53 exon 5 from tumour DNA (**B**). The mutated sequence (**B**) is compared with the wild-type exon 5 sequence from normal control DNA (**A**). Tumour sample (**B**) shows the deletion of one base. The deleted base is indicated in heavy type in **A** or by Δ in **B**. In case no. 20 (**B**), in spite of LOH detected by FISH analysis, the mutated sequence (**B**) shows the presence of a faint normal sequence due to the inevitable presence of small portions of non-tumoral components in the samples. Interphase breast cancer nuclei analysed by FISH (**C**) for the detection of TP53 and chromosome 17 copy number; the green spots correspond to the TP53 gene and the red one to the chromosome 17 pericentromeric region. FISH results show the deletion of TP53 related to the deletion of the whole chromosome 17

tested tumours showed an abnormal SSCP pattern and were all confirmed as mutated by direct DNA sequencing. Table 1 shows the details regarding the genetic changes identified by DNA sequencing analysis.

The position and incidence of the mutations were distributed as follows: three mutations were located in exon 5, three in exon 6, three in exon 7, one in exon 8 and one in intron 6. Eight were missense mutations: seven resulted in an amino acid substitution; the remaining one revealed a neutral polymorphism at codon 213, exon 6, which does not result in a change in amino acid sequence (CGA → CGG, Arg → Arg) (Carbone et al, 1991; Elledge et al, 1993; Berns et al; 1996). Missense mutations included six transitions (one at CpG dinucleotides) and two transversions. Six of the seven (86%) amino acid substitutions detected in our series occurred at mutational hotspot codons (codons 175, 194, 220, 245, 248 and 249) located in evolutionary highly conserved regions (Greenblatt et al, 1994).

Two of the 11 mutations were non-missense mutations, including a 1-bp deletion at codon 176 and a 1-bp insertion at codon 176. Both these situations change the translation frame and lead to the appearance of a premature stop codon, resulting in an early chain termination during translation. Furthermore, in one case, a base substitution was found at 15 bp downstream of the 3′ end of exon 6. This intronic alteration, to the best of our knowledge, should not affect TP53 gene expression.

The observed neutral polymorphism at codon 213 (case no. 29) and intronic base substitution (case no. 1) were not recorded as mutations in the correlations specified below of molecular data with cytological grading, ICC and IA analysis data.

### *Molecular analysis vs cytological grading*

Eight out of the nine (88%) mutated cases and 13 out of the 22 (59%) wild-type cases showed high nuclear grade (G3), indicating a significant association (Table 1).

### *Molecular analysis vs ICC*

p53 immunoreactivity correlated with the type of gene mutation. All but one (83%) of the ICC-positive cases presented a missense mutation that leads to an amino acid change; only one case (no. 6) had a wild-type *p53* gene. The ICC-negative cases included a deletion, an insertion and only one missense mutation. The association between missense mutations and ICC was statistically significant ($P = 0.0007$, Fisher's exact test).

### FISH analysis

Seventeen cases were evaluable for both TP53 and chromosome 17 content, seven only for TP53 content and seven were not evaluable at all. Chromosome 17 pericentromeric signals were detectable in only 17 slides, this may be due to a problem of fixation and/or conservation of the slides before FISH. Moreover, p53 probe was detected with two layers of avidin-FITC as a chromosome 17 α-satellite with only one layer of rhodamine-labelled anti-digoxigenin (ab) to avoid background artifacts.

Four out of 24 cases evaluable for TP53 content showed the deletion of one TP53 allele (cases nos. 19, 20, 25, 28). One of them was also mutated (case no. 19). Nine out of 17 cases evaluable for chromosome 17 content were polysomic and one was monosomic (data not shown).

### DNA (IA)

The analysis was performed on 29 cases. In two cases, insufficient material was available (Table 1). Twenty-six out of twenty-nine cases (89%) were aneuploid, of which three were hypertetraploid, whereas 3 out of 29 (10%) were diploid.

In the aneuploid tumours, MI ranged from 0.32 to 7759.44 (mean 446.13, median 27.45) (data not shown).

© Cancer Research Campaign 1998

### *IA vs molecular analysis*

Comparing ploidy results with molecular data, all cases with TP53 mutations were aneuploid.

### *IA vs MIB1 and cytological grading*

Comparing ploidy results with MIB1 immunostaining, 24 out of 26 (92%) aneuploid cases showed a high MIB1 immunostaining. Furthermore, all aneuploid cases were G3 or G2 with high MIB1 immunostaining (> 30%).

### DISCUSSION

This study shows for the first time, to the best of our knowledge, the feasibility of an extensive structural and quantitative analysis of TP53 on material obtained from FNA. The same material allowed us to perform DNA analysis and compare both molecular, molecular–cytogenetic and DNA content data to a number of routinely used immunocytochemical-based parameters.

Although a statistically significant correlation was observed between p53 overexpression and TP53 mutation ($P = 0.004$), and the observed immunoreactivity pattern fitted fully with the characterized mutation type, the results clearly show a definitively higher informativity of molecular over immunophenotypic analysis. In fact, no ICC reaction was observed in the four cases harbouring TP53 mutations. Two of these false-negative ICC results (case nos. 20 and 26) were generated as a consequence of premature stop codons that determine the synthesis of truncated proteins and thus render ICC detection impossible. In addition, we did not find immunoreactivity in case numbers 30 and 10, despite TP53 missense mutations at codons 248 and 220 respectively. The results observed in case 30 are in agreement with the hypothesis reported by Greenblatt et al (1994), who observed that mutants at codon 248 (Arg→Trp), implying this particular amino acid change, maintain apparent wild-type conformation by antibody analysis but acquire mutant transactivation function. The discrepancy between ICC and molecular results in case number 10 is difficult to explain as the potential functional significance of this mutation has not yet been verified.

In view of all the above, and taking into consideration the patient selection, the failure of immunophenotyping to detect mutations closely mirrors that observed for histology-based material (Dunn et al, 1993; Aas et al, 1996; Sjögren et al, 1996). This observation not only indicates that ICC alone is an insufficient and unreliable indicator of *p53* gene mutations in breast carcinoma, but also points out a significant agreement between the present FNA-based data and those obtained from conventional histological samples.

In one case (no. 6), molecular results did not coincide with p53-positive immunostaining. This might be due to a mutation localized in exons outside the screening area (exons 5–8). Approximately 10–20% of TP53 gene mutations have been found in exons 1–4 or 9–11 (Soussi et al, 1994; Hartmann et al, 1995). However, mutations in other genes coding for proteins involved in the p53 pathway (Momand et al, 1992; Righetti et al, 1996) or the presence of p53-interacting proteins may also contribute to p53 stabilization.

Furthermore, DNA sequencing highlighted that seven out of ten mutations, three of which showed no immunoreactivity, affected the central part of the gene, which includes the zinc-binding domains L2 (codons 163–195) and L3 (codons 236–251).

TP53 molecular and FISH analyses were successfully combined, allowing the detection of four cases with deletion of one TP53 allele; three of them presented, therefore, only one TP53 normal allele and are, in case of a subsequent TP53 mutation, at higher risk of p53 loss of function. The remaining case presented both deletion and mutation (Figure 1), which, with the homozigous mutation, represents the most tumorigénic situation (Solomon et al, 1991). In this particular situation, the significance of the presence of only *p53*-mutated protein in tumour cells has not yet been fully explored. A possible anomalous transactivating activity of *p53*-mutated complexes might confer a different biological behaviour to the affected cells. The present approach could potentially reveal more such cases; further analysis would clarify this point.

The rationale for comparing TP53 alterations with DNA analysis stems from the in vitro and in vivo evidence showing that loss, or inactivation, of TP53 can be associated with unstable tetraploid cells that are predisposed for the development of aneuploid populations (Cross et al, 1995). Although in the present series, 17 DNA aneuploid cases showed no TP53 mutations, indicating the possibility of redundant control mechanisms for genetic stability, it is worth mentioning that all nine cases harbouring TP53 mutations showed aneuploid DNA content, and two out of three hypertetraploid cases belonged to the TP53-mutated cases. Furthermore, the results support a close correlation between aneuploidy, high MIB1 value, high cytological grade and show that MIB1 may usefully complement grading by contributing to a more accurate definition of the grey zone represented by grade II and may positively affect cytological diagnosis (Pinder et al, 1995). In fact, similarly to grade III, all grade II cases with a high MIB1 value fell within the aneuploid group.

In conclusion, molecular analysis appears to be mandatory to acquire more precise information, not only regarding the type of TP53 mutation but also the TP53 genomic status for the discrimination between wild-type–mutant complexes (dominant-negative effect) and mutant–mutant proteins, which could confer different biological properties to the tumour cells; FNA seems to be suitable for this type of study. Complementary DNA analysis stresses the strong predictivity of aneuploidy for grading either represented as grade III or grade II and critically supplemented by ICC-MIB1 assessment.

The results demonstate that PCR-SSCP and sequencing, FISH and DNA image analyses can be simultaneously and successfully performed on FNA material, making FNA a reliable and helpful tool for the prognostic assessment and the therapeutic management of breast cancer patients.

### ACKNOWLEDGEMENTS

This work was supported by grants from AIRC/FIRC (Associazione and Fondazione Italiana per la Ricerca sul Cancro) and partly by CNR (Consiglio Nazionale delle Ricerche) PF 'ACRO'. We thank Mr. Mario Azzini for photographic assistance.

### REFERENCES

Aas T, Børresen AL, Geisler S, Smith-Sørensen B, Johnsen H, Varhaug JE, Akslen LA and Lønning PE (1996) Specific p53 mutations are associated with *de novo* resistance to doxorubicin in breast cancer patients. *Nature Med* **2**: 811–814

Alsabeh R, Wilson CS, Chul WA, Mohammad AV and Battifora H (1996) Expression of bcl-2 by breast cancer: a possible diagnostic application. *Mod Pathol* **9**: 439–444

Bergh J, Norberg T, Sjögren S, Lindgren A and Holmberg L (1995) Complete sequencing of the p53 gene provides prognostic information in breast cancer patients, particularly in relation to adjuvant systemic therapy and radiotherapy. *Nature Med* **1**: 1029–1034

Berns EMJJ, Klijn JGM, Smid M, Van Staveren IL, Look MP, Van Putten WLJ and Foekens JA (1996) TP53 and MYC gene alterations independently predict poor prognosis in breast cancer patients. *Genes Chromosom Cancer* **16**: 170–179

Böcking A, Adler CP, Common HH, Hilgarth M, Granzen B and Auffermann W (1984) Algorithm for a DNA-cytophotometric diagnosis and grading of malignancy. *Anal Quant Cytol* **6**: 1–8

Böcking A, Giroud F and Reith A (1995) Consensus report of the ESACP task force on standardization of diagnostic DNA image cytometry. *Anal Cell Pathol* **8**: 67–74

Cajulis RS, Frias-Hidvegi D, Yu GH and Eggena S (1996) Detection of numerical chromosomal abnormalities by fluorescence in situ hybridization of interphase cell nuclei with chromosome-specific probes on archival cytologic samples. *Diagn Cytopathol* **14**: 178–181

Carbone D, Chiba I and Mitsudomi T (1991) Polymorphism at codon 213 within the p53 gene. *Oncogene* **6**: 1691–1692

Cross SM, Sanchez CA, Morgan CA, Schimke MK, Ramel S, Idzerda RL, Raskind WH and Reid BJ (1995) A p53-dependent mouse spindle checkpoint. *Science* **267**: 1353–1356

Donghi R, Longoni A, Pilotti S, Michieli P, Della Porta G and Pierotti MA (1993) Gene p53 mutations are restricted to poorly differentiated and undifferentiated carcinomas of the thyroid gland. *J Clin Invest* **91**: 1753–1760

Dowell SP, Lane DP and Hall PA (1994) The immunocytochemical detection of p53 protein in cytological specimens: technical considerations. *Cytopathology* **5**: 76–81

Dunn JM, Hastrich DJ, Newcomb P, Webb JCJ, Maitland NJ and Farndon JR (1993) Correlation between p53 mutations and antibody staining in breast carcinoma. *Br J Surg* **80**: 1410–1412

Elledge RM, Fuqua SAW, Clark GM, Pujol P, Allred DC and McGuire WL (1993) Prognostic significance of p53 gene alterations in node-negative breast cancer. *Breast Cancer Res Treat* **26**: 225–235

Fisher ER, Redmond C and Fisher B (1980) Histologic grading of breast cancer. *Pathol Annu* **15**: 239–251

Fricker J (1996) p53 tumour mutations predict response to therapy. *Lancet* **348**: 115

Frigo B, Pilotti S, Zurrida S, Ermellino L, Manzari A and Rilke F (1995) Analysis of estrogen and progesterone receptors on preoperative fine-needle aspirates. *Breast Cancer Res Treat* **33**: 179–184

Greenblatt MS, Bennett WP, Hollstein M and Harris CC (1994) Mutations in the p53 tumor suppressor gene: clues to cancer etiology and molecular pathogenesis. *Cancer Res* **54**: 4855–4878

Hartmann A, Blaszyk H, McGovern RM, Schroeder JJ, Cunningham J, Vries De EMG, Kovach JS and Sommer SS (1995) p53 gene mutation inside and outside of exons 5–8: the patterns differ in breast and other cancers. *Oncogene* **10**: 681–688

Kovach JS, Hartmann A, Blaszyk H, Cunningham J, Schaid D and Sommer SS (1996) Mutation detection by highly sensitive methods indicates that p53 gene mutations in breast cancer can have important prognostic value. *Proc Natl Acad Sci USA* **93**: 1093–1096

Layfield LJ (1992) Can fine-needle aspiration replace open biopsy in the diagnosis of palpable breast lesions? *Am J Clin Pathol* **98**: 145–147

Leong AS-Y (1996) Immunostaining of cytologic specimens. *Am J Clin Pathol* **105**: 139–140

Lichter P, Chang Tang C, Call K, Hermanson G, Evans GA, Housman D and Ward DC (1990) High resolution mapping of human chromosome 11 by in situ hybridization with cosmid clones. *Science* **247**: 64–67

Lowe SW, Ruley HE, Jacks T and Housman DE (1993) p53-dependent apoptosis modulates the cytotoxicity of anticancer agents. *Cell* **74**: 957–967

Lowe SW, Bodis S, Mcclatchey A, Remington L, Ruley HE, Fisher DE, Housman DE and Jacks T (1994) p53 status and the efficacy of cancer therapy in vivo. *Science* **266**: 807–810

Masood S, Frykberg ER, Mclellan GL, Scalapino Mc, Mitchum Dg and Bullard B (1990) Prospective evaluation of radiologically directed fine-needle aspiration biopsy of nonpalpable breast lesions. *Cancer* **66**: 1480–1487

Momand J, Zambetti GP, Olson DC, Georege D and Levine AJ (1992) The mdm-2 oncogene product forms a complex with the p53 protein and inhibits p53-mediated trans activation. *Cell* **69**: 1237–1245

Pinder SE, Wencyk P, Sibbering DM, Bell JA, Elston CW, Nicholson R, Robertson JFR, Blamey RW and Ellis IO (1995) Assessment of the new proliferation marker MIB1 in breast carcinoma using image analysis: associations with other prognostic factors and survival. *Br J Cancer* **71**: 146–149

Ried T, Baldini A, Rand TC and Ward DC (1992) Simultaneous visualization of seven different DNA probes by in situ hybridization using combinatorial fluorescence and digital imaging microscopy. *Proc Natl Acad Sci USA* **89**: 1388–1392

Righetti SC, Della Torre G, Pilotti S, Ménard S, Ottone F, Colnaghi Mi, Pierotti MA, Lavarino C, Cornarotti M, Oriana S, Böhm S, Bresciani GL, Spatti G and Zunino F (1996) A comparative study of p53 gene mutations, protein accumulation, and response to cisplatin-based chemotherapy in advanced ovarian carcinoma. *Cancer Res* **56**: 689–693

Sauter G, Feichter G, Torhorst J, Moch H, Novotna H, Wagner U, Dürmüller U and Waldman FM (1996) Fluorescence *in situ* hybridization for detecting ErbB-2 amplification in breast tumor fine needle aspiration biopsies. *Acta Cytol* **40**: 164–173

Sjögren S, Inganäs M, Norberg T, Lindgren A, Nordgren H, Holmberg L and Bergh J (1996) The p53 gene in breast cancer: prognostic value of complementary DNA sequencing versus immunohistochemistry. *J Natl Cancer Inst* **88**: 173–182

Solomon E, Borrow J and Goddard A (1991) Chromosome aberrations and cancer. *Science* **254**: 1153–1160

Soussi T, Legros Y, Lubin R, Ory K and Schlichtholz B (1994) Multifactorial analysis of p53 alteration in human cancer: a review. *Int J Cancer* **57**: 1–9

Thomas PA, Vazquez MF and Waisman J (1990) Comparison of fine-needle aspiration and frozen sections of palpable mammary lesions. *Mod Pathol* **3**: 570–574

UICC International Union Against Cancer (1992) *TNM Classification of Malignant Tumors,* 4th edn. Hermanek P and Sobin LH. (eds), pp. 103–109. Springer: Berlin

Wahl AF, Donaldson Kl, Fairchild C, Lee FYF, Foster SA, Demers GW and Galloway DA (1996) Loss of normal p53 function confers sensitization to Taxol by increasing G2/M arrest and apoptosis. *Nature Med* **2**: 72–79

© Cancer Research Campaign 1998